# Temporal changes and determinants of childhood nutritional status in Kenya and Zambia

**DOI:** 10.1186/s41043-017-0095-z

**Published:** 2017-06-05

**Authors:** Daniel Hoffman, Thomas Cacciola, Pamela Barrios, James Simon

**Affiliations:** 1 Department of Nutritional Sciences, Rutgers University, New Brunswick, NJ USA; 2 New Jersey Institute for Food, Nutrition, and Health, Center for Childhood Nutrition Education and Research, Program in International Nutrition, Rutgers University, 61 Dudley Road, New Brunswick, NJ 08901 USA; 3 Department of Plant Biology, Rutgers University, New Brunswick, NJ USA

**Keywords:** Stunting, Wasting, Nutritional status, Children, Kenya, Zambia, Dietary diversity

## Abstract

**Background:**

The prevalence of undernutrition is decreasing in many parts of the developing world, but challenges remain in many countries. The objective of this study was to determine factors influencing childhood nutrition status in Kenya and Zambia. The objective of this study is to determine factors associated with temporal changes in childhood nutritional status in two countries in sub-Saharan Africa.

**Methods:**

Data from national demographic and health surveys from the World Bank for Kenya (1998–2009) and Zambia (1996–2014) were used to select the youngest child of each household with complete data for all variables studied. Multiple linear regression analyses were used for data from 2902 and 11,335 children from Kenya and Zambia, respectively, in each year to determine the relationship between social and economic factors and measures of nutritional status, including wasting, stunting, and overweight.

**Results:**

There was a decreased prevalence of stunting (35% in Kenya and 40% in Zambia), while the prevalence of wasting was unchanged (6–8% in both countries). From 1998 to 2009, there was a protective effect against stunting for wealthier families and households with electricity, for both countries. Finally, better educated mothers were less likely to have stunted children and girls were less likely to be stunted than boys.

**Conclusions:**

Based on the data analyzed, there was a higher risk of stunting in both Kenya and Zambia, for those with lower literacy, less education, no electricity, living in rural areas, no formal toilet, no car ownership, and those with an overall lower wealth index. Improving the education of mothers was also a significant determinant in improving the nutritional status of children in Kenya and Zambia.

More broad-based efforts to reduce the prevalence of undernutrition need to focus on reducing the prevalence of undernutrition without promoting excess weight gain. Future economic advances need to consider integrated approaches to improving economic standings of households without increasing the risk for overnutrition.

## Background

The prevalence of obesity is a major public health problem in most developed and many transitional countries and has also contributed to the “double burden of disease”, the coexistence of both obesity and underweight [1–5]. The double burden is not necessarily a reflection of competing problems [6] and the fact that excess weight gain is now co-existent with low-weight underscores the need to better understand how chronic nutrition problems change over time and within specific socio-geographic regions. To that end, it is of interest to better understand what factors influence temporal changes in nutritional status, especially in less developed regions that have yet to experience the double burden of disease, such as Sub-Saharan Africa. Therefore, the focus of this paper is to describe the current state of childhood nutrition in Kenya and Zambia and determine factors associated with temporal changes in childhood nutritional status.

In terms of childhood nutrition, a high prevalence of chronic malnutrition has been reported in both urban and rural settings throughout Africa [7–13]. For example, a high percentage of children are stunted, underweight, or wasted, in Nairobi, Kenya, but most especially within informal urban settlements [11, 14]. For example, in the Dagoretti Division of Nairobi, 24.5% of children aged 4–11 were stunted, 14.9% were underweight, and 9.7% were wasted [14]. At the same time, more boys than girls are undernourished; however, there are some conflicting results with different trends emerging between separate regions [11, 14–16]. Specifically, a higher percentage of boys were stunted compared to girls [14]. In Kibera, an informal settlement in Nairobi, one survey of 1310 children aged 6–59 months found that 47.0% of children were stunted (severe stunting in 23.4% of the children), 11.8% were underweight (severe underweight in 3.1%), and 2.6% were wasted (severe wasting in 0.6%) [11]. Moreover, girls were more likely than boys to be wasted at 3 years of age compared to boys. Such gender differences in these data may arise from a number of cultural or economic factors that favor one gender over the other. These studies highlight the fact that poor nutrition is most prevalent in impoverished and marginalized areas, but the cross-sectional nature of the research does not allow for broader conclusions regarding how social determinants of nutritional status may change with time.

With regards to undernutrition, the prevalence of stunting or wasting varies across Sub-Saharan Africa and even within rural regions of Kenya. One recent study reported that fetal growth restriction and poor sanitation are the primary predictors of stunting in many parts of the world, but most particular for Sub-Saharan Africa [7]. Within country data are more illustrative, such that in the rural Bondo district of Kenya, 30% of children under the age of 5 were stunted (12% severely stunted), 4% were wasted (1% severely wasted), and 20% were underweight (5% severely underweight) with height and weight deficits most prevalent for children aged 18–23 months [10]. Meanwhile, in the Suba district, children between the ages of 11 and 16 years had the highest percentages of undernourished subjects and the most severe undernutrition with boys more likely to be stunted and underweight compared to girls. One study of malnutrition rural Kenya found that among children under the age of 60 months, there was a higher prevalence of malnutrition among girls compared to boys. In addition, girls also tended to have lower overall energy intake compared to boys. In particular, of the 629 subjects surveyed in the Mwingi and Makueni districts, stunting, underweight, and wasting were all more prevalent among girls [16]. Thus, gender differences may confound other determinants of nutritional status, emphasizing the need for more comprehensive research on factors that influence childhood nutritional status.

In Zambia, one study of children from the Samfya district found that food intake of infants and toddlers was insufficient such that total energy, calcium, iron, and vitamin A were below recommended daily intake for both infants and toddlers, while infants were also below the recommended intake for protein [9]. Moreover, weaning foods consumed by toddlers were found to be inadequate as well, increasing the risk for continued nutritional deficits during childhood [9]. A study from the Chroma district reported poor nutritional status in a sample of 388 children aged 24-59 months and among children aged 12-23 months, only 40% were adequately nourished [13]. Finally, one study in Zambia focused on adults in the Katete district and reported that lower self-perceived socioeconomic status was related to a lower adult BMI in the sample of 254 men and women [17].

In summary, it is clear that undernutrition continues to be a serious problem that persists in these two countries of Eastern Africa [18–20]. However, while these studies have consistently reported a high prevalence of childhood undernutrition, they often do not extend the research to determine how nutritional status is affected by other factors, such as urbanization, education, and maternal autonomy. Therefore, additional research is need to better understand how various social and economic conditions can be modified to promote better nutritional status of children and adults in both countries. The objective of this paper is to determine socio-economic factors that influence childhood nutritional status in a temporal setting using nationally representative data from Kenya and Zambia.

## Methods

Using data from the Health Nutrition and Population Statistics of national demographic and health surveys (DHS) at the World Bank [20], the prevalence of stunting and wasting was calculated using available years for both Kenya and Zambia. The sampling framework for DHS is fully covered in the manual for DHS data collection [21]. Briefly, the ministry responsible for DHS can submit data collected only if the survey follows key principles explained in detail in the DHS manual. Such principles include the use of an existing sampling frame that provides full coverage of the target population (such as households with children) and is conducted using a random design with a sample size consistent with the manual. In addition, households sampled must conform to the selection criteria and strict confidentiality is maintained. Datasets were extracted from the World Bank website for each country and year studied. Statistical analyses were conducted on the datasets after the deletion of missing values, implausible values, and only respondents with all available data for each variable studied were included. After data cleaning, the final dataset studied for each country included more than 2500 children (ages birth to 4 years) for each year in both Kenya and Zambia. For each outcome of interest, social and economic factors that may influence each was analyzed using stepwise linear regression to best determine how such factors are modified by year of each survey. Using this method allowed for us to determine how specific factors that are associated with nutritional status vary as time progresses, especially in light of the fact that each country has experienced consistent economic growth of 5% of greater since the mid-1990s 20, 21. All data were analyzed using SPSS version 22 (IBM SPSS Statistics, NY, USA) and statistical significance was set a *p* < 0.05.

### Nutritional status

The prevalence of stunting and wasting in Kenya and Zambia was calculated according to the WHO guidelines [22] in which stunting was defined as a height-for-age Z-score (HAZ) < −2.00 and wasting was defined as a weight-for-height Z-score (WHZ) < −2.00. Overweight was defined as WHZ < 2.00 and BMI percentile for age above 85%. According to the conceptual framework of poverty proposed by UNICEF [23], nutritional status is the outcome of a complex hierarchy of factors that begins with direct exposure to quality diet and health care and extends to more indirect interactions with social and economic infrastructure that contribute to a myriad of socio-environmental factors that ultimately contribute to a child’s nutritional status.

Multivariate logistic regression analyses were used to determine how social and economic factors contribute to risk of stunting and wasting, as well as potential changes across time. Specifically, the main outcomes of stunting and wasting were entered as the dependent variables in two models for each country. Known risk factors for these conditions were entered as independent variables, including wealth index, number of household members, rural or urban setting, type of toilet, maternal age, maternal educational status, and age and sex of the child. Backward stepwise analyses were conducted and only the statistically significant independent variables were included in each year analyzed for each country. This was the preferred method to determine if specific variables differed in terms of influencing the nutritional status of the child over the time period studied.

## Results

A summary of the temporal changes in childhood nutritional status is presented in Table 1. The prevalence of stunting in Kenya averaged 35% for the years analyzed while the prevalence in Zambia decreased from 50% in 1996 to 40% in 2014. Wasting remained a less prevalent condition with an average of 7% of Kenyan and 6% of Zambian children suffering from wasting. At the same time, approximately 6% of Kenyan and Zambian children are classified as overweight (Table 2 and Table 3).

**Table 1:** Nutritional status (%) of children in Kenya and Zambia in selected years of available data

	Kenya			Zambia			
Year	1998	2003	2009	1996	2002	2007	2014
*N*	5478	5150	5088	5478	5150	5088	11335
Wasted (WHZ < −2.0)	37	34.7	34.5	50.3	53.7	44.3	39.9
Stunted (HAZ < −2.0)	7.5	6.8	8.1	5.4	5.9	5.9	6.3
Wasted (BMI percentile)	7.7	5.6	5.2	6.1	5.3	8.3	5.8
Overweight (WHZ > 2.0)	9	7	6.4	8.1	7.7	10.7	7.1

**Table 2:** Demographic characteristics of households surveyed for Kenya between 1998 and 2009

	1998					2003					2009				
Characteristics	*N*	% of total	% Stunted	% Wasted	% OW	*N*	% of total	% Stunted	% Wasted	% OW	*N*	% of total	% Stunted	% Wasted	% OW
Type of place of residence															
Urban	418	14.3	29.2	6.2	8.6	1076	23.7	27.9	5.8	8.2	1182	23.2	27.3	6.3	5.4
Rural	2503	85.7	38.4	7.8	7.6	3462	76.3	36.8	7.2	4.7	3914	76.8	36.6	8.7	5.1
Source of drinking water															
Piped	747	25.6	32.9	6.7	9.8	1266	27.9	27.4	4.7	7.8	1392	27.3	29.8	7.4	5.5
Well	731	25.0	36.7	7.4	8.1	859	18.9	33.4	9.1	4.5	1372	26.9	34.5	8.1	5.0
Surface water	1360	46.6	39.9	8.2	6.4	2125	46.8	40.2	6.8	4.6	2143	42.1	37.8	8.7	4.8
Rainwater	33	1.1	36.4	3.0	6.1	288	6.3	30.2	9.4	5.9	189	3.7	30.2	7.4	9.0
Has electricity															
No	2668	91.3	38.7	7.6	7.1	3948	87.0	36.8	7.3	4.6	4302	84.4	36.6	8.7	5.0
Yes	244	8.4	19.3	6.6	14.3	589	13.0	20.7	3.7	11.7	792	15.5	23.0	4.8	6.6
Toilet type															
Flush toilet	171	5.9	24.0	7.0	12.9	421	9.3	19.2	4.0	11.9	484	9.5	23.3	5.0	8.1
Pit toilet	2219	76.0	36.0	7.2	8.2	3062	67.5	34.8	4.7	5.2	3320	65.1	33.6	5.9	5.1
None	517	17.7	46.0	9.5	4.1	1031	22.7	40.6	14.1	4.1	1255	24.6	40.9	14.8	4.5
Has car															
No	2826	96.7	37.6	7.7	7.4	4342	95.7	35.6	7.0	5.3	4924	96.6	35.0	8.2	5.1
Yes	87	3.0	20.7	3.4	16.1	187	4.1	14.4	2.7	11.2	166	3.3	19.9	4.2	7.8
Has television															
No	2603	89.1	39.0	7.8	7.2	3773	83.1	37.5	7.4	4.8	3961	77.7	37.8	9.0	5.1
Yes	306	10.5	20.6	5.6	12.1	759	16.7	21.1	3.8	9.5	1129	22.2	22.5	5.2	5.6
Literacy															
Reads easily	1746	59.8	33.3	6.0	8.5	2942	64.8	31.9	4.3	6.4	3061	60.1	31.5	5.1	5.5
Reads with difficulty	671	23.0	39.0	10.1	6.9	359	7.9	39.8	3.3	4.2	672	13.2	40.0	8.6	6.0
Cannot read	496	17.0	48.2	9.7	6.3	1228	27.1	40.1	13.9	4.0	1317	25.8	38.6	14.5	4.3
Highest educational level															
No education	335	11.5	47.2	9.0	5.4	826	18.2	39.1	17.1	3.9	1042	20.4	38.4	16.7	4.5
Primary	1863	63.8	39.8	8.3	7.8	2691	59.3	38.4	5.1	5.4	2908	57.1	37.0	6.3	4.8
Secondary	672	23.0	26.3	5.2	8.2	825	18.2	23.6	3.3	6.8	878	17.2	26.1	4.7	6.8
Higher	51	1.7	11.8	2.0	13.7	196	4.3	12.8	3.1	10.2	268	5.3	19.0	5.6	7.1
Head of household															
Male	2148	73.5	37.2	7.3	8.2	3435	75.7	35.0	6.9	5.6	3627	71.2	33.9	8.0	5.2
Female	773	26.5	36.7	8.3	6.2	1103	24.3	33.9	6.5	5.3	1469	28.8	35.9	8.4	5.3
Child age															
0	1801	37.0	20.0	9.6	11.7	1145	25.2	29.0	6.1	7.7	1161	22.8	20.1	11.4	10.2
1	1020	34.9	45.1	8.0	5.2	971	21.4	39.1	6.8	4.0	1002	19.7	45.0	7.4	5.7
2	820	28.1	49.6	4.1	5.6	886	19.5	37.8	6.3	5.8	1058	20.8	43.2	6.8	3.8
3		0.0				842	18.6	34.8	8.4	5.0	984	19.3	34.0	5.7	2.4
4		0.0				694	15.3	33.9	6.8	4.6	891	17.5	31.4	9.0	2.8
Age of mother															
Under 20	414	14.2	36.7	7.2	9.2	503	11.1	36.2	7.6	7.0	531	10.4	32.8	11.3	6.0
20–29	1549	53.0	37.5	7.4	7.7	2296	50.6	34.0	6.6	5.1	2557	50.2	33.7	7.5	5.5
30+	958	32.8	36.5	7.8	7.1	1739	38.3	35.2	6.9	5.8	2008	39.4	35.9	8.1	4.6
Employment^a^															
Not currently employed	1414	48.4	37.8	7.3	7.9	1816	40.0	33.6	8.3	6.2	2283	44.8	35.1	10.6	5.5
Currently employed	1506	51.6	36.5	7.8	7.6	2718	59.9	35.5	5.8	5.2	2812	55.2	33.9	6.1	5.0
Household members															
2–6 members	1756	60.1	37.2	7.9	8.3	2880	63.5	34.2	6.1	6.1	3281	64.4	33.0	7.5	5.9
7+ members	1165	39.9	36.9	7.0	6.9	1658	36.5	35.6	8.0	4.6	1815	35.6	37.1	9.2	4.0
Wealth index															
Poorest (1)	702	24.0	46.6	8.8	5.0	1124	24.8	43.4	11.7	3.8	1479	29.0	42.1	13.5	5.0
Poorer (2)	647	22.1	41.0	10.0	7.4	898	19.8	37.5	6.9	3.8	931	18.3	37.7	6.7	4.4
Middle (3)	567	19.4	35.1	5.8	9.3	843	18.6	33.9	4.5	5.6	850	16.7	32.7	6.1	4.9
Richer (4)	555	19.0	33.5	5.6	8.1	736	16.2	32.3	5.7	5.6	844	16.6	30.0	6.4	5.1
Richest (5)	450	15.4	23.6	6.4	9.8	937	20.6	24.1	3.9	9.3	992	19.5	25.3	4.7	6.6
Sex of child															
Male	1453	49.7	42.3	8.0	8.0	2273	50.1	36.0	7.3	5.3	2598	51.0	37.0	8.9	5.3
Female	1468	50.3	31.9	7.1	7.4	2265	49.9	33.4	6.4	5.8	2498	49.0	31.8	7.3	5.1

Stunted defined by HAZ score, wasted and overweight by WHZ

^a^Employment of mother

**Table 3:** Demographic characteristics of households surveyed for Zambia between 1996 and 2014

	1996					2001–02					2007					2014				
Characteristics	*N*	% of total	% Stunted	% Wasted	% OW	*N*	% of total	% Stunted	% Wasted	% OW	N	% of total	% Stunted	% Wasted	% OW	*N*	% of total	% Stunted	% Wasted	% OW
Type of place of residence																				
Urban	1820	33.0	41.1	3.7	5.6	1295	15.1	42.7	6.3	5.6	1602	31.3	38.3	5.7	7.7	4129	36.2	35.9	6.4	6.2
Rural	3697	67.0	54.8	6.2	6.4	3870	74.9	57.4	5.8	5.2	3519	68.7	47.0	6.0	8.5	7278	63.8	42.1	6.3	5.6
Source of drinking water																				
Piped	1640	29.7	40.4	3.9	5.5	1241	24.0	42.5	6.0	5.6	1235	24.1	36.5	5.7	7.5	2646	23.2	32.2	6.7	6.7
Well	2910	52.7	54.1	5.9	6.5	2816	54.5	55.9	5.6	4.5	2649	51.7	45.4	6.5	7.5	6609	57.9	40.9	6.4	5.4
Surface water	925	16.8	55.8	6.3	6.4	1095	21.2	60.6	6.7	6.9	1079	21.1	50.4	4.6	11.7	2032	17.8	46.3	5.7	6.0
Rainwater						13	0.3	53.8	0.0	0.0	158	3.1	43.7	5.7	3.2	120	1.1	46.7	5.8	5.8
Has electricity																				
No	4636	84.0	53.6	5.8	6.1	4529	87.7	56.3	5.9	5.2	4418	86.3	46.3	6.0	8.4	9338	81.9	42.0	6.4	5.6
Yes	871	15.8	33.1	3.0	5.9	633	12.3	34.6	6.3	5.8	703	13.7	31.6	5.5	7.1	2044	17.9	30.0	6.0	6.7
Toilet type																				
Flush toilet	841	15.2	34.2	4.0	5.5	506	9.8	37.5	7.1	6.1	439	8.6	31.7	4.6	6.4	964	8.5	26.1	6.7	6.8
Pit toilet	2958	53.6	52.9	5.3	6.3	3071	59.5	53.7	5.9	5.2	3293	64.3	45.3	5.4	8.4	8454	74.1	40.8	6.5	5.7
None	1689	30.6	53.4	6.0	6.1	1578	30.6	58.9	5.6	5.2	1361	26.6	45.3	7.3	8.2	1949	17.1	42.6	5.4	5.8
Has car																				
No	5390	97.7	50.9	5.4	6.1	5062	98.0	54.2	5.9	5.3	5030	98.2	44.7	5.9	8.3	10803	94.7	40.6	6.4	5.7
Yes	113	2.0	21.2	6.2	8.0	89	1.7	28.1	5.6	4.5	91	1.8	23.1	7.7	6.6	591	5.2	25.7	5.8	6.8
Has television																				
No	4575	82.9	53.8	5.8	6.2	4412	85.4	56.3	6.1	5.2	3996	78.0	47.0	6.2	8.7	7985	70.0	42.6	6.8	5.5
Yes	930	16.9	33.2	3.4	5.6	751	14.5	38.1	5.3	5.6	1122	21.9	34.4	5.0	6.8	3417	30.0	33.5	5.2	6.5
Literacy																				
Reads easily	2248	40.7	45.0	42.0	5.3	2215	42.9	47.6	5.4	4.2	2165	42.3	41.1	5.0	8.1	5265	46.2	35.7	5.8	5.9
Reads with difficulty	1141	20.7	50.5	5.4	7.1	482	9.3	55.4	4.6	5.8	597	11.7	50.3	7.2	9.7	1078	9.5	42.2	7.9	5.7
Cannot read	2122	38.5	55.7	6.6	6.5	2416	46.8	59.0	6.7	6.3	2239	43.7	45.9	6.3	8.2	4990	43.7	43.8	6.6	5.7
Highest educational level																				
No education	832	15.1	57.8	6.5	7.2	775	15.0	59.0	6.3	5.9	677	13.2	44.0	7.4	6.6	1289	11.3	43.8	7.3	5.7
Primary	3578	64.9	52.1	5.7	5.6	3324	64.4	56.3	6.0	5.4	3214	62.8	47.1	5.7	8.9	6404	56.1	42.4	6.2	5.5
Secondary	1014	18.4	41.1	3.5	6.9	988	19.1	43.3	5.8	3.9	1119	21.9	38.2	5.5	7.7	3314	29.1	36.1	6.3	6.0
Higher	91	1.6	12.1	4.4	6.6	78	1.5	23.1	3.8	9.0	111	2.2	24.3	6.3	6.3	393	3.4	17.0	5.9	8.9
Head of household																				
Male	4646	84.2	49.5	5.6	6.2	4394	85.1	53.7	5.8	5.6	4240	82.8	44.2	5.7	8.3	9306	81.6	39.7	6.1	5.7
Female	871	15.8	54.3	4.4	5.9	771	14.9	53.4	6.2	3.5	881	17.2	44.6	6.9	7.9	2101	18.4	40.6	7.2	6.1
Child age																				
0	1376	24.9	26.7	9.9	6.9	1275	24.7	50.1	5.9	7.4	1176	23.0	25.3	9.4	14.5	2423	21.2	23.7	8.9	12.4
1	1241	22.5	54.5	7.7	3.6	1179	22.8	59.1	6.7	4.2	1137	22.2	51.5	6.2	6.5	2384	20.9	48.7	6.5	5.2
2	1130	20.5	65.0	2.6	6.7	1031	20.0	57.1	3.5	5.6	1015	19.8	52.7	5.4	6.4	2289	20.1	50.3	5.5	3.9
3	950	17.2	60.3	2.0	7.8	896	17.3	48.5	8.7	4.9	975	19.0	48.7	3.0	7.1	2192	19.2	41.7	5.2	3.9
4	820	14.9	51.7	2.0	5.7	784	15.2	52.7	5.0	3.3	818	16.0	45.8	4.6	5.5	2119	18.6	35.3	5.2	3.0
Age of mother																				
Under 20	690	12.5	50.1	7.0	5.2	692	13.4	54.3	8.7	6.1	491	9.6	43.4	6.7	6.9	1257	11.0	43.0	6.0	7.8
20-29	2725	49.4	50.3	5.3	6.0	2544	49.3	53.5	5.6	5.4	2562	50.0	45.7	5.8	8.4	5178	45.4	40.0	6.3	5.7
30+	2102	38.1	50.2	4.9	6.6	1929	37.3	53.7	5.4	4.8	2068	40.4	42.7	5.9	8.4	4972	43.6	39.0	6.4	5.4
Employment_a_		0.0	0.0	0.0	0.0															
Not currently employed	2665	48.3	48.6	5.5	6.4	2059	39.9	51.4	6.5	5.7	2440	47.6	44.6	5.7	9.3	4808	42.1	38.6	5.9	5.9
Currently employed	2851	51.7	51.9	5.2	5.8	3104	60.1	55.2	5.6	5.0	2676	52.3	43.9	6.1	7.3	6599	57.9	40.8	6.6	5.7
Household members																				
2–6 members	2587	46.9	53.7	5.5	5.5	2827	54.7	54.4	6.7	5.6	3054	59.6	46.2	5.8	8.6	6130	53.7	41.1	6.2	6.1
7+ members	2930	53.1	47.3	5.3	6.6	2338	45.3	52.8	5.0	4.9	2067	40.4	41.5	6.0	7.7	5277	46.3	38.5	6.5	5.5
Wealth Index		0.0	0.0	0.0	0.0															
Poorest (1)	1643	29.8	58.1	7.2	5.9	1277	24.7	60.5	5.9	4.7	1140	22.3	47.4	7.1	9.1	2757	24.2	47.2	7.1	5.3
Poorer (2)	993	18.0	55.1	5.8	6.4	1181	22.9	59.1	5.8	5.3	1131	22.1	49.6	5.7	7.9	2755	24.2	42.4	6.5	4.8
Middle (3)	1112	20.2	52.0	4.7	7.0	1227	23.8	54.1	6.4	5.6	1188	23.2	46.9	5.3	9.8	2605	22.8	38.9	5.6	6.8
Richer (4)	954	17.3	44.8	4.2	4.8	870	16.8	48.6	5.5	4.8	1030	20.1	39.2	6.0	6.8	1905	16.7	36.6	6.2	5.9
Richest (5)	815	14.8	32.8	3.4	6.0	610	11.8	35.2	6.2	6.2	632	12.3	32.4	4.9	6.8	1385	12.1	26.6	5.9	6.7
Sex of child																				
Male	2688	48.7	51.6	6.2	6.2	2585	50.0	54.6	6.2	5.3	2512	49.1	47.4	6.7	8.6	5721	50.2	42.2	6.6	6.3
Female	2829	51.3	49.1	4.6	6.0	2580	50.0	47.2	5.7	5.3	2609	50.9	41.3	5.1	8.0	5686	49.8	37.5	6.0	5.3

Stunted defined by HAZ score, wasted and overweight by WHZ

^a^Employment of mother

Results of the multivariate logistic regression analyses are summarized in Tables 4 and 5 for stunting and wasting, respectively. With regards to socio-economic factors in Kenya, the higher the wealth index of a family, the lower the risk of having a stunted child for all years analyzed. For 1998 and 2008 only, those households that reported having electricity were less likely to have a stunted child compared to those without electricity. There were no significant results for the number of family members, household setting, or type of toilet for any of the years analyzed.

**Table 4:** Multivariate logistic regression model for risk factors in relation to stunting in children in Kenya (1998–2009) and Zambia (1996–2014)

		Kenya						Zambia							
		1998		2003		2008–09		1996		2001–02		2007		2013–14	
Household characteristics		OR (CI)	*P*	OR (CI)	*P*	OR (CI)	*P*	OR (CI)	*P*	OR (CI)	*P*	OR (CI)	*P*	OR (CI)	*P*
Wealth index quintile	Poorest	.	.	.	.	.	.	.	.	.	.	.	.	.	.
	Poor	0.82 (0.65 -1.03)	0.09	0.81 (0.69 - 0.97)	0.02	0.82 (0.68 - 0.98)	0.03	0.91 (0.77 - 1.07)	0.27	0.95 (0.81 - 1.12)	0.54	1.09 (0.93 - 1.30)	0.29	0.84 (0.75 - 0.93)	0.00
	Middle	0.68 (0.53 - 0.86)	0.00	0.70 (0.58 - 0.85)	0.00	0.68 (0.56 - 0.82)	0.00	0.72 (0.61 - 0.86)	0.00	0.79 (0.67 - 0.93)	0.00	0.98 (0.83 - 1.16)	0.83	0.72 (0.64 - 0.81)	0.00
	Rich	0.64 (0.50 - 0.83)	0.00	0.69 (0.57 - 0.85)	0.00	0.63 (0.52 - 0.77)	0.00	0.56 (0.46 - 0.68)	0.00	0.72 (0.60 - 0.87)	0.00	0.74 (0.62 - 0.89)	0.00	0.67 (0.58 - 0.78)	0.00
	Richest	0.54 (0.38 - 0.76)	0.00	0.53 (0.43 - 0.65)	0.00	0.66 (0.51 - 0.85)	0.00	0.50 (0.31 - 0.81)	0.00	0.73 (0.46 - 1.15)	0.17	0.84 (0.59 - 1.20)	0.33	0.50 (0.41 - 0.61)	0.00
Household members	2-6	.	.	.	.	.	.	.	.	.	.	.	.	.	.
	7+	.	.	.	.	.	.	0.87 (0.78 - 0.98)	0.03	.	.	.	.	.	.
Setting	Urban vs Rural	.	.	.	.	.	.	.	.	.	.	.	.	0.84 (0.75 - 0.94)	0.00
Toilet Type	Flush	.	.	.	.	.	.	.	.	.	.	.	.	.	.
	Pit	.	.	.	.	.	.	1.01 (0.72 - 1.42)	0.95	.	.	.	.	.	.
	None	.	.	.	.	.	.	0.80 (0.55 -1.15)	0.23	.	.	.	.	.	.
Electricity	No vs Yes	0.63 (0.41 - 0.99)	0.04	.	.	0.75 (0.58 - 0.97)	0.03	0.69 (0.50 - 0.95)	0.02	0.58 (0.38 - 0.88)	0.01	0.69 (0.51 - 0.95)	0.02	.	.
Family Characteristics															
Mother Age	Years	0.98 (0.97 - 0.99)	0.01	.	.	.	.	0.99 (0.98 - 1.00)	0.04	.	.	0.98 (0.98 - 0.99)	0.00	0.99 (0.98 - 0.99)	0.00
Educational Level (mother)	None	.	.	.	.	.	.	.	.	.	.	.	.	.	.
	Primary	0.74 (0.57 - 0.96)	0.02	1.11 (0.93 - 1.31)	0.25	1.12 (0.95 - 1.32)	0.17	0.88 (0.75 - 1.04)	0.13	0.98 (0.84 - 1.16)	0.84	1.15 (0.96 - 1.36)	0.12	0.98 (0.87 -1.11)	0.76
	Secondary	0.46 (0.34 - 0.63)	0.00	0.62 (0.49 - 0.78)	0.00	0.79 (0.63 - 0.98)	0.03	0.74 (0.60 - 0.92)	0.00	0.73 (0.59 - 0.90)	0.00	0.93 (0.75 - 1.14)	0.47	0.87 (0.75 - 1.00)	0.05
	Higher	0.22 (0.09 - 0.54)	0.00	0.34 (0.22 - 0.55)	0.00	0.58 (0.40 - 0.85)	0.01	0.13 (0.06 - 0.27)	0.00	0.39 (0.22 - 0.69)	0.00	0.65 (0.39- 1.06)	0.09	0.43 (0.32 - 0.59)	0.00
Child age	Months	1.06 (1.06 - 1.07)	0.00	.	.	1.01 (1.00 - 1.01)	0.00	1.03 (1.02 - 1.03)	0.00	.	.	1.02 (1.01 - 1.02)	0.00	1 (1.00 - 1.01)	0.00
Child Sex	Boy vs Girl	0.62 (0.53 - 0.72)	0.00	0.88 (0.78 - 0.99)	0.04	0.76 (0.69 - 0.87)	0.00	0.86 (0.77 - 0.96)	0.01	.	.	0.76 (0.68 - 0.85)	0.00	0.82 (0.76 - 0.88)	0.00

*Adjusted for all variables in the table, which were selected by forward stepwise regression

**Table 5:** Multivariate logistic regression model for risk factors in relation to wasting* in children in Kenya (1998–2009) and Zambia (1996–2014)

		Kenya						Zambia							
		1998		2003		2008–09		1996		2001–02		2007		2013–14	
Household characteristics		OR (CI)	*P*	OR (CI)	*P*	OR (CI)	*P*	OR (CI)	*P*	OR (CI)	*P*	OR (CI)	*P*	OR (CI)	*P*
Wealth index quintile	Poorest	.	.	.	.	.	.	.	.	.	.	.	.	.	.
	Poor	1.14 (0.79 - 1.65)	0.48	.	.	.	.	.	.	.	.	.	.	.	.
	Middle	0.63 (0.41 - 0.97)	0.04	.	.	.	.	.	.	.	.	.	.	.	.
	Rich	0.59 (0.38 - 0.93)	0.02	.	.	.	.	.	.	.	.	.	.	.	.
	Richest	0.69 (0.44 - 1.10)	0.11	.	.	.	.	.	.	.	.	.	.	.	.
Household members	2-6	.	.	.	.	.	.	.	.	.	.	.	.	.	.
	7+	.	.	.	.	.	.	.	.	0.74 (0.58 - 0.94)	0.01	.	.	.	.
Setting	Urban vs Rural	.	.	.	.	.	.	1.65 (1.24 - 2.18)	0.00	.	.	.	.	.	.
Toilet Type	Flush	.	.	.	.	.	.	.	.	.	.	.	.	.	.
	Pit	.	.	1.02 (0.59 - 1.76)	0.95	1.26 (0.79 - 2.02)	0.33	.	.	.	.	1.22 (0.76 - 1.99)	0.42	.	.
	None	.	.	1.82 (1.02 - 3.26)	0.04	2.29 (1.39 - 3.79)	0.00	.	.	.	.	1.70 (1.04 - 2.79)	0.04	.	.
Family Characteristics															
Mother Age	Years	.	.	.	.	.	.	.	.	.	.	.	.	1.01 (1.00 - 1.02)	0.05
Educational Level (mother)	None	.	.	.	.	.	.	.	.	.	.	.		.	.
	Primary	.	.	0.37 (0.27 - 0.50)	0.00	0.46 (0.36 - 0.60)	0.00	.	.	.	.	.	.	.	.
	Secondary	.	.	0.26 (0.16 - 0.42)	0.00	0.40 (0.27 - 0.60)	0.00	.	.	.	.	.	.	.	.
	Higher	.	.	0.25 (0.10 - 0.61)	0.00	0.57 (0.31 - 1.07)	0.08	.	.	.	.	.	.	.	.
Currently employed (mother)	No vs Yes	.	.	.	.	0.72 (0.58 - 0.89)	0.00	.	.	.	.	.	.	.	.
Child age	Months	0.97 (0.96 - 0.99)	0.00	.	.	0.99 (0.99 - 0.99)	0.00	0.96 (0.95 - 0.97)	0.00	.	.	0.98 (0.97 - 0.99)	0.00	0.99 (0.98 - 0.99)	0.00
Child Sex	Boy vs Girl	.	.	.	.	.	.	0.75 (0.59 - 0.96)	0.02	.	.	0.76 (0.60 - 0.96)	0.02	.	.

*Adjusted for all variables in the table, which were selected by forward stepwise regression

Regarding maternal-child characteristics, more educated mothers were 50–80% less likely to have a stunted child compared to mothers with no or primary education only. However, the degree to which education reduced the odds of having a stunted child was not as great in 2009 compared to 1998. Older children were more likely to be stunted than younger children in 1998 and 2008, but not in other years. Finally, for all 3 years analyzed, girls were less likely to be stunted compared to boys.

The relationship between the socio-economic factors and stunting in Zambia were similar to what was found in Kenya. However, in Zambia, a large family decreased the odds of having a stunted child (1996) as did living in a rural, compared to an urban, area (2014). Also, having a flush versus pit or no toilet was protective against stunting in 1991, but not in other years. Households with electricity was protective through 2007. The results for the influence of maternal-child characteristics on stunting in Zambia were similar to Kenya such that mothers’ age had a borderline protective effect and maternal education was protective, but only in 1996 and 2001.

For wasting, in Kenya, there was a protective effect of wealth on the odds of having a wasted child in 1998 only. Not having a toilet in the household, either pit or flush, increased the odds of having a wasted child by more than 80% in 2003 and over 200% in 2009. As was reported for stunting, the higher education reported by a mother decreased the odds of having a wasted child in 2003 and 2009. Finally, maternal employed decreased the odds of having a wasted child in 2009 only.

In Zambia, the relationships between socio-economic factors and wasting differed from those in Kenya. Briefly, as was reported for stunting, a large family reduced the odds of having a wasted child (2001) while living in a rural area increased the odds (1996). Not having a pit or flush toilet in 2007 increased the odds of having a wasted child, as did being a boy in 1996 and 2007.

Summarizing the most salient outcomes, we found that the risk of stunting was higher for those with lower literacy, less education, no electricity, living in rural areas, no formal toilet, no car ownership, and those with an overall lower wealth index. This trend was consistent for both Kenya and Zambia and from year to year of available data (1998 to 2009 for Kenya and 1996 to 2014 for Zambia). Results for wasting, a condition that is a reflection of the daily nutrient intake and acute disease state, there were similar trends, but less pronounced differences between levels of each socio-economic factor.

## Discussion

Undernutrition continues to be a major public health issue in Sub-Saharan Africa [23, 24], including both Kenya and Zambia [9, 25]. In fact, a number of studies have examined the nutritional status and dietary intake of children and adults in each country [11, 14]. While many of these studies have reported insufficient nutrient intake and low food security for those living in Kenya and Zambia [10, 11, 14, 26], the larger context of social factors that are associated with nutritional status and how such relationships change over time need to also be recognized. Briefly, using nationally representative data over a period of ten years in Kenya and Zambia, we found that the prevalence of stunting has not changed since 1998 in Kenya, but has decreased by 20% in Zambia since 1996. For both countries, the key predictors of chronic nutritional deficiencies (i.e., stunting) were maternal education, elements of higher socio-economic status (e.g., electricity, modern toilet, television, and piped water), while those for acute nutritional insults (i.e., wasting) included higher wealth index, type of toilet, level of maternal education, and the sex of child for some years in Zambia.

The results of our analyses are consistent with studies from other parts of Sub-Saharan Africa. In particular, as was reported by Danaei and colleagues, poor fetal growth and unsanitary conditions are the major factors predicting stunting in children under the age of 5 years [7]. This study is complemented by an economic analysis of stunting in which it was estimated stunting in Sub-Saharan African results in over $18 million in lost educational attainment [27]. Thus, given the complex nature of factors that influence growth as well as the profound effects on economics, it is imperative to understand how various programs may reduce the prevalence of stunting. Nabwera et al. reported that intensive health and nutrition interventions decreased the prevalence of undernutrition by 50% in Gambia, but that more comprehensive and sustainable programs are needed to have a more significant and lasting impact on childhood health [28]. In addition, apparent differences in nutritional status between boys and girls illustrates that there may be factors that favor higher dietary intake among one gender over the other, differences in work or physical activity patterns between the two genders, or cultural preferences for one gender over the other. Simply, any of these factors could promote healthy growth or poor growth depending on the soci-economic context in which the child is exposed.

Compared to other cross-sectional studies from each country, our data clearly demonstrate that factors influencing diet and nutritional status in Kenya and Zambia have not changed appreciably in the past 20 years. For example, one study of 1000 homes in Nairobi reported that 85% of those households surveyed were food insecure and 50% were severely food insecure [29]. The relationship between dietary, social, and environmental factors and obesity were examined in another study. A sample of 1008 women from across Kenya was used for the study, which involved anthropometric measurements and 24-h dietary recall interviews. The researchers found that overweight and obesity were highly prevalent among Kenyan women, with 43.3% of women overweight or obese. The highest prevalence of overweight and obesity occurred in women in urban areas within the high-income group [30]. The time of year was related to food security in Kenya based on the effects of the rainy and dry seasons. Using data from Meru County, it was determined that intake of energy, protein, iron, zinc, calcium, and folate increased in the rainy season and that household food security increased from the dry to the rainy season. Data was obtained from 525 households using interviews of mothers or caregivers [31]. A program involving educational intervention was found to increase the diversity of diets in a sample of 207 households. The intervention took place in Bondo and Teso South sub counties and consisted of training and cooking demonstrations for caregivers, which increased the children’s dietary diversity scores and the caregiver’s nutrition knowledge scores [32]. Surveys of adults and households illustrate the fact that many households are food insecure and reveal relationships between the nutritional status of individuals and various non-dietary factors.

Aside from those factors analyzed in this study, many other socio-demographic factors may account for the results presented, including culture, geography, maternal autonomy, and food aid [12, 33]. It is clear that specific cultural groups or geographical areas within each country have varying degrees of vulnerability to food insecurity that may influence childhood nutritional status. One study of dietary intake patterns of three ethnic groups in Kenya (Luo, Kamba, and Maasai) found that the Maasai and Kamba were vulnerable to food insecurity compared to the Luo [34]. Specifically, the prevalence of underweight, a key indicator of food insecurity, was 13.7% for the Luo, 20.5% for the Kamba, and 24.2% for the Maasai [34]. Regarding maternal autonomy, the more independent a mother is to makes decisions related to health care, education, food, and have an independent source of income , the more likely her children are to be properly nourished [35–38]. In the Siaya, Kisumu, and Busia districts of Kenya, a high prevalence of childhood undernutrition was attributed to low maternal independence and high martial discord as childcare is not only affected by a mother’s direct actions with her child, but also through her social relationships with others because of the assistance that others provide [39]. In terms of food aid, a study in the Yatta district investigated the effectiveness of using local foods to improve the nutritional status of children and found that a small food ration provided to families resulted in a lower prevalence of wasting and underweight compared to control families [40]. Similar to our results, two studies in urban settlements of Nairobi (Korogocho and Viwandani) found that a higher maternal educational status was associated with higher nutritional status of her child [35, 41]. Therefore, based on our results and those of others, there has not been an appreciable change in the factors associated with nutritional status, suggesting potential avenues for nutrition interventions to combat undernutrition in these countries.

As with any study using nationally representative data and questionnaires, there are limitations that merit discussion. First, diet was assessed using different methodologies between waves of national data collection that could introduce bias or inconsistency between years studied. Second, it is unclear from DHS data exactly who was trained to measure nutritional status in each wave of data collection for each country. There is also limited information on quality control checks. Nonetheless, DHS data are considered reliable given that they are collected using established methods and have rigid oversight by the ministries responsible for administering the surveys. Still, the data should be treated with some degree of caution, but solid conclusions may be drawn given the established methods and near universal acceptance of these data as being nationally representative by major government and non-governmental organizations.

While the sub-Saharan countries of Kenya and Zambia have yet to begin the “nutrition transition” [42], it is clear that undernutrition is becoming less of a problem than before periods of economic growth. However, considering the analyses that demonstrate the intractable relationships between socio-economic factors and risk of poor nutritional status, future economic advances need to consider integrated approaches to improving economic standings of households without increasing the risk for overnutrition. Specifically, advancing maternal education and general household wealth without the introduction of processed foods and foods that are not part of the traditional diet are paramount. Research to improve the dietary diversity, one that includes nutrition dense and culturally acceptable foods, such as African-indigenous vegetables, is one example of improving economic development and nutrition without the risk of excess weight gain. Rigorous studies of how such work can impact diet and health are needed and should be part of interdisciplinary approaches to improving health and nutrition in developing countries.

## Conclusions

In conclusion, based on nationally representative household data, the risk for stunting in both Kenya and Zambia was higher for those with lower literacy, less education, no electricity, living in rural areas, no formal toilet, no car ownership, and those with an overall lower wealth index. Therefore, improving the education of mothers was also a significant determinant in improving the nutritional status of children in Kenya and Zambia. In addition, the need for more broad-based efforts to reduce the prevalence of undernutrition that focus on reducing the prevalence of undernutrition without promoting excess weight gain is great. As such, future economic advances need to consider integrated approaches to improving economic standings of households without increasing the risk for overnutrition.

## Abbreviations

DHS: Demographic household surveys
HAZ: Height for age Z-score
WHZ: Weight for height Z-score
